# An obstetric sphincter injury risk identification system (OSIRIS): is this a clinically useful tool?

**DOI:** 10.1007/s00192-016-3125-2

**Published:** 2016-09-02

**Authors:** Sara S. Webb, Karla Hemming, Madhi Y. Khalfaoui, Tine Brink Henriksen, Sara Kindberg, Stine Stensgaard, Christine Kettle, Khaled M. K. Ismail

**Affiliations:** 1 Institute of Metabolism and Systems Research, College of Medical and Dental Sciences, University of Birmingham, Birmingham, UK; 2 Delivery Suite, Birmingham Women’s NHS Foundation Trust, Mindelsohn Way, Edgbaston, Birmingham, B15 2TG UK; 3 Institute of Applied Health Research, University of Birmingham, Birmingham, UK; 4 School of surgery, North Western Deanery, Manchester, UK; 5 Perinatal Epidemiology Research Unit, Aarhus University Hospital, Aarhus, Denmark; 6 Staffordshire University, Stafford, UK

**Keywords:** Cohort study, OASIS, Risk factors, Predictor variables, Prognostic model

## Abstract

**Introduction and hypothesis:**

To establish the contribution of maternal, fetal and intrapartum factors to the risk of incidence of obstetric anal sphincter injuries (OASIS) and assess the feasibility of an OASIS risk prediction model based on variables available to clinicians prior to birth.

**Methods:**

This was a population-based, retrospective cohort study using single-site data from the birth database of Aarhus University Hospital, Denmark. The participants were all women who had a singleton vaginal birth during the period 1989 to 2006. Univariate and multivariate logistic regression analyses were performed using multiple imputations for missing data and internally validated using bootstrap methods. The main outcome measures were the contributions of maternal, fetal and intrapartum events to the incidence of OASIS.

**Results:**

A total of 71,469 women met the inclusion criteria, of whom 1,754 (2.45 %) sustained OASIS. In the multivariate analysis of variables known prior to birth, maternal age 20 – 30 years (OR 1.65, 95 % CI 1.44 – 1.89) and ≥30 years (OR 1.60, 95 % CI 1.39 – 1.85), occipitoposterior fetal position (OR 1.34, 95 % CI 1.06 – 1.70), induction/augmentation of labour (OR 1.46, 95 % CI 1.32 – 1.62), and suspected macrosomia (OR 2.20, 95 % CI 1.97 – 2.45) were independent significant predictors of OASIS, with increasing parity conferring a significant protective effect. The ‘prebirth variable’ model showed a 95 % sensitivity and a 24 % specificity in predicting OASIS with 1 % probability, and a 3 % sensitivity and a 99 % specificity in predicting OASIS with a 10 % probability.

**Conclusions:**

Our model identified several significant OASIS risk factors that are known prior to actual birth. The prognostic model shows potential for ruling out OASIS (high sensitivity with a low risk cut-off value), but is not useful for ruling in the event.

**Electronic supplementary material:**

The online version of this article (doi:10.1007/s00192-016-3125-2) contains supplementary material, which is available to authorized users.

## Introduction

Obstetric anal sphincter injuries (OASIS) are serious complications of vaginal birth with a reported incidence throughout Europe and globally from 1 % to 10 % [1, 2]. Recent UK data demonstrate a steadily rising incidence over the past 12 years, which, in the absence of changes in major risk factors, is possibly due to improved detection [2]. OASIS is one of the most significant risk factors for anal incontinence in young women [3], long-term dyspareunia and perineal pain [4]. Despite optimal primary repair, approximately 39 % of women who sustain an OASIS will suffer from anal incontinence [5].

With the wealth of available population-based databases, prediction models are being developed to assist in clinical decision making [6]. Several large population-based cohort studies have identified various independent risk factors for sustaining primary or recurrent OASIS. These include nulliparity, increased birth weight, operative vaginal delivery, persistent occipitoposterior position, prolonged pushing phase, induction of labour, use of epidural analgesia and shoulder dystocia [7–10], although findings across studies are contradictory. However, there is a paucity of information relating to the impact of risk factors that can only be known prior to the actual birth both on the likelihood of OASIS occurring de novo and on the usefulness of such information for counselling women as to their potential individualized risk of sustaining a primary OASIS.

The aims of this study were twofold. The primary aim was to explore both the burden of the contribution of each variable and the combined effects of multiple factors on the risk of OASIS in singleton pregnancies. The secondary aim was to develop an OASIS prognostic model using variables known only prior to birth and to evaluate its feasibility for accurately predicting the individual risk of OASIS in singleton pregnancies to provide women and clinicians with information for making informed decisions about intrapartum management and mode of birth.

## Materials and methods

This was a retrospective population-based cohort study using the birth cohort database of Aarhus University Hospital, Denmark. All women who had singleton pregnancies and a vaginal birth during the period 1989 – 2006 were included. For the purposes of our analysis the classification of OASIS in the current RCOG Green-top Guideline was used [11], and all degrees of perineal tears involving the anal sphincter complex (third-degree tears 3a, 3b and 3c, and fourth-degree tears) documented in our cohort were grouped into one variable (OASIS). The information contained in the database included: antenatal demographics including maternal age, parity and body mass index (BMI); intrapartum events including use of oxytocin for induction/augmentation of labour, intrapartum fetal position, duration of pushing phase in minutes, operative vaginal delivery, epidural anaesthesia and mediolateral episiotomy; and postnatal data including gestational age at birth, actual fetal birth weight and fetal head circumference. Information relating to previous history of OASIS was not recorded in the database.

The study sample was first summarized by comparing women who sustained and women who did not sustain OASIS, reporting means and standard deviations (medians and interquartile ranges for skewed variables and numbers and percentages for categorical variables). The two groups were compared using *t* tests for continuous data, and the Mann-Whitney *U* test was used for continuous skewed variables and the chi-squared test for categorical variables.

A comprehensive stepwise procedure was used to select independent factors predicting OASIS. This included the evaluation of two logistic regression models reporting odds ratios (OR) and 95 % confidence intervals (CI). The first model (obstetric sphincter injury risk identification system, OSIRIS – All Variables model) was developed using all independent predictive factors available (antenatal, intrapartum and postnatal data). This allowed consideration of all factors available in the database. However, as some of these factors are only known after birth they may not necessarily be useful as part of a clinical prediction model. Consequently, a second logistic regression model (OSIRIS – Prebirth Variables model), reporting OR and 95 % CI and including predictive factors that could only be determined or planned before the actual birth of the baby, was developed. These variables included maternal age, gestational age, BMI, parity, induction/augmentation of labour, fetal position, and the presence of fetal macrosomia. In our database, the actual birth weight was documented for each birth, but no assumption as to the presence of macrosomia was recorded prior to delivery. Although ultrasonographic biometric indices and the accuracy of estimated fetal weight remain low, they are improving [12]; therefore we felt it was reasonable to assume that the presence of macrosomia was a determinable variable prior to delivery. Hence, we included the widely accepted and pragmatic threshold of 4,000 g birth weight as a predictive variable in the second model.

Stepwise selection methods were used to identify independent predictive variables. This was done independently for each set of the two prediction models developed. This consisted of a forward stepwise selection procedure (with a *P* value for addition set at 0.05 and for removal set at 0.1) complemented with a sensitivity check involving the implementation of a backwards stepwise selection procedure. Given the large size of the dataset and the likelihood of statistical significance being obtained, a further criterion for selecting a predictor was that it should obtain a prespecified clinical importance (OR greater than 1.1 or less than 0.9; for continuous variables the OR related to the unit SD change).

Multiple imputations using chained equations with predictive mean matching was used as all predictors had at least some data missing. Ten multiple imputations were used and continuous variables were analysed as linear predictors within the multiple imputations but included as categorical or continuous covariates (*z* scores) within the stepwise regression models, to allow selection of the most important variables.

The discriminatory abilities of the models produced (to distinguish between those who will and those who will not have an adverse outcome) were evaluated using the C statistic [13] (area under the ROC curve). The larger the C statistic the greater the degree of separation in a prognostic model. To reduce over-optimism in model estimates due to potential over-fitting in the model development data, bootstrapping was used to produce C statistics and model coefficients that were adjusted for over-optimism. Model calibration was also assessed using the Hosmer-Lemeshow goodness-of-fit test by plotting predicted outcome probabilities by decile group against the observed outcome probabilities in the same decile group.

Participants were classified as at risk or not at risk of sustaining OASIS based on their estimated risk from the OSIRIS – Prebirth Variables model. The OASIS probability cut-off values of 1 %, 2 %, 5 % and 10 % were used. Several variations of the risks associated with these cut-off values were compared, and for each set of cut-off values, the sensitivity, specificity and positive and negative predictive values in natural frequencies were determined. No formal power calculations were performed. However, general guidelines suggested a minimum of ten events per variable considered in the development of the model [14]. As there were 1,754 events, investigating a maximum of 17 predictor variables provided ample power. All analyses were carried out using Stata 12 [15].

## Results

A total of 71,469 women had a vaginal birth in the unit during the study period, and of these 1,754 (2.45 %) sustained OASIS, an incidence slightly lower than the overall UK rate [16]. Table 1 shows the univariate analysis of the baseline maternal and labour characteristics of the included women. Maternal age was comparable between the women who did and did not sustain OASIS (mean ± SD age 29.9 ± 4.6 years and 29.8 ± 4.1 years, respectively). BMI was also similar between the two groups, with about 70 % of the women having a BMI ranging from 18.5 to 24.99 kg/m^2^. There were, however, significant differences (between those with and without an OASIS) in parity, fetal malposition, use of induction/augmentation, episiotomy, instrumental delivery, use of epidural anaesthesia, birth weight ≥4,000 g, duration of pushing phase, head circumference, gestational age and actual birth weight.

**Table 1:** Baseline characteristics of the patients

Characteristics	No OASIS event (*N* = 69,715)	OASIS event (*N* = 1,754)	OR (95 % CI)	*P* value^e^
Maternal characteristics				
Age (years), mean ± SD	29.9 ± 4.6	29.8 ± 4.1	0.99 (0.99 – 1.01)	0.649
BMI (kg/m^2^)^a^				
<18.5	3,479 (6.3 %)	85 (5.8 %)	1	
18.5 – 24.99	39,392 (71.7 %)	1,040 (70.8 %)	1.08 (0.86 – 1.35)	0.497
25 – 29.99	9,107 (16.6 %)	277 (18.8 %)	1.24 (0.97 – 1.59)	0.081
≥30	2,937 (5.4 %)	68 (4.6 %)	0.95 (0.67 – 1.31)	0.744
Parity^b^				
0	27,802 (48.8 %)	1,147 (75.6 %)	1	
1	20,311 (35.7 %)	310 (20.4 %)	0.37 (0.33 – 0.42)	0.000
2	6,691 (11.8 %)	54 (3.6 %)	0.20 (0.15 – 0.26)	0.000
3 or more	2,120 (3.7 %)	6 (0.4 %)	0.07 (0.03 – 0.15)	0.000
Labour characteristics				
Fetal position				
Occipitoanterior	55,509 (93.3 %)	1,532 (92.9 %)	1	
Occipitoposterior	1,949 (3.3 %)	82 (5.0 %)	1.52 (1.22 – 1.91)	0.000
Other	2,030 (3.4 %)	36 (2.2 %)	0.64 (0.46 – 0.90)	0.009
Induction/augmentation^d^	34,372 (49.3 %)	1,132 (64.5 %)	1.87 (1.70 – 2.07)	0.000
Episiotomy (all mediolateral)^c^	12,091 (17.7 %)	71 (4.1 %)	0.20 (0.16 – 0.25)	0.000
Instrumental^c^	6,828 (9.8 %)	602 (34.4 %)	4.74 (4.28 – 5.25)	0.000
Epidural anaesthesia^c^	4,351 (26.8 %)	204 (33.2 %)	1.36 (1.14 – 1.61)	0.000
Macrosomia^d^	12,501 (18.0 %)	559 (32.0 %)	2.14 (1.93 – 2.37)	0.000
Duration of pushing phase (min)^c^				
<30	41,704 (63.3 %)	601 (36.0 %)	1	
30 – 59	15,685 (23.8 %)	569 (34.0 %)	2.52 (2.24 – 2.83)	0.000
60 – 119	7,754 (11.8 %)	450 (26.9 %)	4.03 (3.55 – 4.56)	0.000
≥120	793 (1.2 %)	51 (3.0 %)	4.46 (3.32 – 5.99)	0.000
Infant characteristics				
Head circumference (cm), mean ± SD^c^	35.0 ± 1.9	35.6 ± 1.5	1.08 (1.06 – 1.10)	0.000
Gestational age (weeks), median (IQR)	40 (39 – 41)	40 (39 – 41)	1.20 (1.16 – 1.24)	0.000
Weight (kg), median (IQR)^c^	3.50 (3.18 – 3.86)	3.74 (3.42 – 4.07)	2.27 (2.08 – 2.49)	0.000

Values are number (percentage) unless otherwise stated. For continuous variables ORs are the means and variance standardized (*z* scores)

*IQR* interquartile range, *SD* standard deviation

^a^BMI is reported for 56,385 patients

^b^Parity is reported for 58,441 patients

^c^Available after delivery only

^d^Assuming suspected macrosomia available before delivery (information available from actual birth weight); and that induction is usually decided before birth

^e^The *t* test was used for continuous variables (with the Mann-Whitney *U* test for skewed data), and the chi-squared test for categorical variables

In the multivariate analysis of all significant factors (OASIS – All Variables model) induction/augmentation of labour (OR 1.40, 95 % CI 1.26 – 1.55), pushing for more than 30 min with the effect increasing with the duration of the active second stage and the largest effect seen in those pushing for 120 min or more (OR 2.69, 95 % CI 1.97 – 3.68), head circumference ≥37 cm (OR 1.20, 95 % CI 1.01 – 1.42), and actual fetal weight (OR 1.71, 95 % CI 1.60 – 1.84) remained significant risk factors for sustaining OASIS, whereas mediolateral episiotomy had a protective effect (OR 0.11, 95 % CI 0.09 – 0.14; Table 2).

**Table 2:** Adjusted odds ratios of OASIS variables after multiple imputation

Characteristics	OASIS – All Variables model		OASIS – Prebirth Variables model	
	Adjusted OR (95 % CI)	*P* value	Adjusted OR (95 % CI)	*P* value
Maternal characteristics				
Age (years)				
≤20			1	
20 – 30			1.65 (1.44 – 1.89)	0.000
≥30			1.60 (1.39 – 1.85)	0.000
BMI (kg/m^2^)				
<18.5				
18.5 – 24.99				
25 – 29.99				
≥30				
Parity				
0	1		1	
1	0.42 (0.36 – 0.48)	0.000	0.36 (0.32 – 0.41)	0.000
2	0.23 (0.18 – 0.30)	0.000	0.20 (0.16 – 0.26)	0.000
3 or more	0.11 (0.05 – 0.21)	0.000	0.09 (0.05 – 0.18)	0.000
Labour characteristics				
Fetal position				
Occipitoanterior			1	
Occipitoposterior			1.34 (1.06 – 1.70)	0.013
Other			0.73 (0.53 – 1.02)	0.068
Induction/augmentation^a^	1.40 (1.26 – 1.55)	0.000	1.46 (1.32 – 1.62)	0.000
Episiotomy (all mediolateral)^b^	0.11 (0.09 – 0.14)	0.000		
Instrumental^b^				
Epidural anaesthesia^b^				
Macrosomia^a^			2.20 (1.97 – 2.45)	0.000
Duration of pushing phase (min)^b^				
<30	1			
30 – 59	1.65 (1.44 – 1.88)	0.000		
60 – 119	2.45 (2.11 – 2.84)	0.000		
≥120	2.69 (1.97 – 3.68)	0.000		
Infant characteristics				
Head circumference ()cm^b^				
≤34	1			
35 – 36	1.11 (0.98 – 1.27)	0.103		
≥37	1.20 (1.01 – 1.42)	0.041		
Gestational age (weeks)			1.27 (1.20 – 1,35)	0.000
Weight (kg), *z* score	1.71 (1.60 – 1.84)	0.000		0.000
ROC (C statistic)	0.774 (0.764 – 0.785)		0.709 (0.697 – 0.721)	

For continuous variables ORs are the means and variance standardized (*z* scores)

^a^Assuming suspected macrosomia available before delivery (information available from actual birth weight); and that induction is usually decided before birth

^b^Available after delivery

However, in the multivariate analysis of only those variables known prior to birth (OSIRIS – Prebirth Variables model) maternal age 20 – 30 years vs. <20 years (OR 1.65, 95 % CI 1.44 – 1.89) and ≥30 years vs. <20 years (OR 1.60, 95 % CI 1.39 – 1.85), occipitoposterior fetal position (OR 1.34, 95 % CI 1.06 – 1.70), induction/augmentation of labour (OR 1.46, 95 % CI 1.32 – 1.62), suspected macrosomia based on the assumption that babies with a birth weight of ≥4,000 g would have been accurately suspected antenatally (OR 2.20, 95 % CI 1.97 – 2.46) all remained as significant predictors of OASIS (Table 2). Both multivariate regression models highlighted the protective effect of increasing parity against OASIS. In the OSIRIS – Prebirth Variables model, women with three or more previous births were least likely to suffer OASIS (OR 0.09, 95 % CI 0.05 – 0.18), but even one previous delivery conferred a significant protective effect (OR 0.36, 95 % CI 0.32 – 0.41; Table 2).

In internal validation the C statistic was 0.77 (95 % CI 0.76 – 0.79) for the OSIRIS – All Variables model, and 0.71 (95 % CI 0.70 – 0.72) for the OSIRIS – Prebirth Variables model. The latter model also showed good calibration (not shown) throughout the risk range. The internally validated bootstrapped multiple imputation results (Table 2) were similar to the main results (Table S1).

**Fig. 1:**
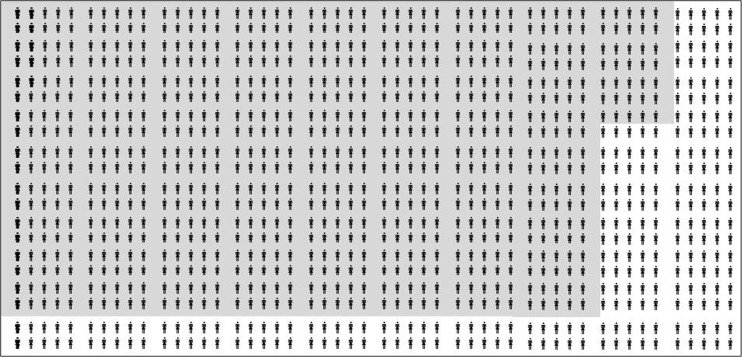
Discriminatory ability of the OASIS – Prebirth Variables model at a 1 % OASIS probability cut-off value. Each ‘woman’ in the figure represents one woman counselled for risk of sustaining OASIS, and of these women, 25 will sustain OASIS (*bold*) and 975 will not (*not bold*). *Grey-shaded cells* indicate women deemed ‘at risk’. *Unshaded cells* indicate women deemed ‘not at risk’

**Fig. 2:**
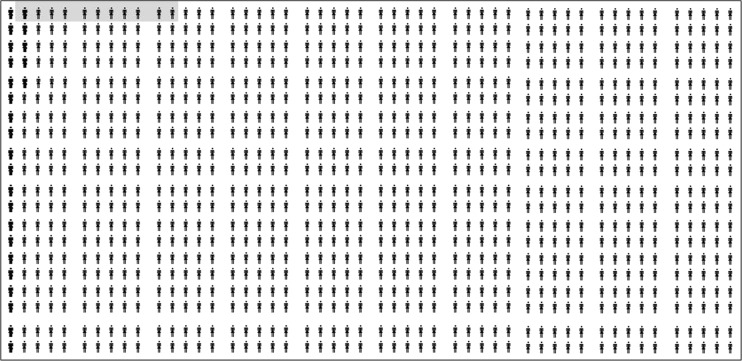
Discriminatory ability of the OASIS – Prebirth Variables model at a 10 % OASIS probability cut-off value. Each ‘woman’ in the figure represents one woman counselled for risk of sustaining OASIS, and of these women, 25 will sustain OASIS (*bold*) and 975 will not (*not bold*). *Grey-shaded cells* indicate women deemed ‘at risk’. *Unshaded cells* indicate women deemed ‘not at risk’

The discriminatory ability of the OSIRIS – Prebirth Variables model was further evaluated by determining the sensitivity and specificity for various probability cut-off values (Table 3). Taking the risk of OASIS as 2.5 % (as observed in our data), applying a risk prediction model cut-off value of 1 % (i.e. any woman with a predicted risk of 1 % or greater was assumed to be at risk of sustaining OASIS during vaginal birth) would ensure that 24 out of the 25 women at a higher risk of sustaining OASIS would be identified. However, this would have a high false-positive rate potentially causing unnecessary anxiety and interventions in 765 women (Fig. 1). Conversely, increasing the cut-off value to 10 % would improve specificity, with the number of women deemed at risk of OASIS reduced to 11. However, this must be weighed against the expected reduction in sensitivity whereby 24 women of the 25 at risk of OASIS would be ‘missed’ (Fig. 2). Consequently, the OSIRIS – Prebirth Variables model is not clinically useful for ruling in OASIS as it would categorize both women who have a few risk factors and those who have all possible risk factors as high risk.

**Table 3:** Sensitivity, specificity, positive and negative predictive values of OASIS pre-birth variables model

	OASIS probability cut-off value			
	1 %^a^	2 %	5 %	10 %^b^
Sensitivity (%)	95	77	23	3
Specificity (%)	24	52	93	99
Positive predictive value (%)	3	4	7	8
Negative predictive value (%)	99	99	98	98
Number of women	1,000	1,000	1,000	1,000
Number with OASIS	25	25	25	25
Number deemed at risk	765	487	74	11
Number “identified”	24	19	6	1
Number deemed not at risk	235	513	926	989
Number “missed”	1	6	19	24

^a^Figure 1

^b^Figure 2

## Discussion

Our study identified several factors that are significant predictors of OASIS, but several of these changed following the multivariate analyses for both models. The factor with the highest contribution to the risk of OASIS when all significant variables were taken into account (OSIRIS – All Variables model) was the duration of the pushing phase, a result not consistently supported by other reports [17, 18], possibly because the recorded duration of the second stage depends on when its onset was determined by vaginal examination. Current intrapartum care NICE guidelines in the UK advise limiting the active second stage in nulliparous and multiparous women to 2 h and 3 h, respectively [19]. Our finding suggests that these recommended limits may be one of the factors contributing to the rising incidence of OASIS in the UK. Nonetheless, it is important to recognize that early interventions to reduce the duration of the second stage can potentially increase operative deliveries. In the OSIRIS – Prebirth Variables model, suspected fetal macrosomia showed the highest association with the risk of OASIS, a finding that has indeed been consistently reported by other researchers [20, 21].

 Some variables were identified as significant in both models, for example the use of oxytocin for induction or augmentation of labour. A similar association has previously been reported by Poen et al. [9]. Therefore, it would be prudent to consider this risk when counselling women who might wish to have an early induction to mitigate the impact of birth weight on OASIS. Maternal age and fetal occipitoposterior position during labour were significantly associated with the risk of OASIS only in our OSIRIS – Prebirth Variables model. The association of these variables with OASIS is supported by the findings of other studies [7, 8, 10]. This study also identified some factors that were significantly associated with a reduction in the risk of OASIS. Multiparity was consistently shown to have such a protective effect in both OSIRIS models despite the fact that parity in our database related to number of deliveries in general rather than the number of previous vaginal deliveries. Interestingly, the use of episiotomy was shown to have a significant protective effect when all variables were considered. There is discrepancy in the reported literature with regard to the association between episiotomy and OASIS [8, 22–25]. This discrepancy is probably a reflection of the difference in types of episiotomies assessed or the variation in the cutting angle of non-midline episiotomies [7, 26].

The main strength of our study lies in the large database used in the analysis that provided a wide variety of factors enabling the development of a clinically meaningful model that excluded variables unknown until after delivery. However, we recognize that the study had several limitations. Because of the retrospective nature of the data, it was not possible to assess the accuracy of perineal trauma assessment, to determine the risk of occult tears, to determine if oxytocin had been used for induction or augmentation, to obtain information about the previous history of OASIS, or to determine if intrapartum interventions known to modify the risk of OASIS, such as manual perineal support [27, 28] or warm compresses [29], had been used. The study period was also long. The study was started before publication in July 2001 of the first edition of the RCOG Green-top Guideline which introduced and recommended standardized classification and repair management of OASIS [30]. Thus because of inconsistent classification, before this date it is possible that perineal trauma involving the anal sphincters was not categorized under what is currently considered OASIS. Finally, the database used in this study did not have estimated fetal weight or suspected macrosomia as an antepartum variable, and hence for this variable we assumed that all babies who had a birth weight of ≥4,000 g could have been suspected antenatally as macrosomic either on clinical abdominal examination or on ultrasonography. Although current research is showing an improvement in the detection of fetuses weighing ≥4,000 g in the 2 weeks prior to delivery, maternal factors such as raised BMI affect the accuracy of such assessments [12]. To mitigate the weakness resulting from the inaccuracy of clinical examination or ultrasound scan, we opted to use the dichotomized variable of suspected macrosomia rather than estimated fetal weight.

Although we were able to prove the feasibility of a statistically robust prognostic model for predicting the individual risk of OASIS using demographic and obstetric factors known prior to birth, we were not able to demonstrate its projected usefulness in a clinical setting to rule in OASIS. Indeed, the OSIRIS Prebirth Variables model would categorize both women who have a few risk factors and those who have all possible risk factors as high risk. Undoubtedly, this high false-positive rate could lead to undue anxiety and potentially and unnecessary interventions. Nevertheless, this model seems to be good at ruling out OASIS; however, the clinical utility of this feature requires further investigation.

## Electronic supplementary material

Below is the link to the electronic supplementary material.

Table S1 (DOC 52 kb)
