# Supplementary material for: An obstetric sphincter injury risk identification system (OSIRIS): is this a clinically useful tool?
Source: Int Urogynecol J. 2016 Sep 2;28(3):367–74. doi: 10.1007/s00192-016-3125-2 (PMC5331086; doi:10.1007/s00192-016-3125-2)
Supplement: Supplementary file 1 — (DOC 52 kb) [file 192_2016_3125_MOESM1_ESM.doc]

| **Table S1** Adjusted odds ratios of OASIS variables before multiple imputation | | | | |
| --- | --- | --- | --- | --- |
|  | **OASIS**  **All Variables model** | | **OASIS**  **Pre-birth Variables Model** | |
| Characteristics | Adjusted OR (95% CI) | P-value | Adjusted OR (95% CI) | P-value |
| **Maternal characteristics** |  |  |  |  |
| Age |  |  |  |  |
| <=20 years |  |  | 1 |  |
| 20-30 years |  |  | 1.68 (1.45, 1.94) | 0.000 |
| ≥ 30 years |  |  | 1.62 (1.39, 1.88) | 0.000 |
| BMI |  |  |  |  |
| <18.5 |  |  |  |  |
| 18.5 – 24.99 |  |  |  |  |
| 25 – 29.99 |  |  |  |  |
| ≥30 |  |  |  |  |
| Parity |  |  |  |  |
| 0 births | 1 |  | 1 |  |
| 1 birth | 0.42 (0.36, 0.49) | 0.000 | 0.36 (0.32, 0.41) | 0.000 |
| 2 births | 0.22 (0.17, 0.29) | 0.000 | 0.19 (0.14, 0.25) | 0.000 |
| 3 or more births | 0.12 (0.06, 0.24) | 0.000 | 0.11 (0.06, 0.20) | 0.000 |
| **Labour characteristics** |  |  |  |  |
| Fetal position |  |  |  |  |
| Occipito-Anterior |  |  | 1 |  |
| Occipito-Posterior |  |  | 1.39 (1.10, 1.74) | 0.005 |
| Other |  |  | 0.73 (0.52, 1.02) | 0.064 |
| Induction/Augmentation^ | 1.41 (1.27, 1.56) | 0.000 | 1.45 (1.31, 1.61) | 0.000 |
| Episiotomy (all mediolateral)* | 0.11 (0.09. 0.14) | 0.000 |  |  |
| Instrumental* |  |  |  |  |
| Epidural* |  |  |  |  |
| Macrosomia^ |  |  | 2.20 (1.97, 2.46) | 0.000 |
| Length of pushing phase* |  |  |  |  |
| <30 minutes | 1 |  |  |  |
| 30-59 minutes | 1.63 (1.43, 1.86) | 0.000 |  |  |
| 60-119 minutes | 2.40 (2.08, 2.77) | 0.000 |  |  |
| ≥ 120 minutes | 2.65 (1.96, 3.60) | 0.000 |  |  |
| **Infant characteristics** |  |  |  |  |
| Head circumference* |  |  |  |  |
| <=34 cm | 1 |  |  |  |
| 35-36 cm | 1.11 (0.98, 1.26) | 0.111 |  |  |
| ≥ 37cm | 1.19 (1.01, 1.40) | 0.041 |  |  |
| Gestational age, weeks |  |  | 1.27 (1.19, 1.36) |  |
| Weight, kg (z-score) | 1.71 (1.60, 1.83) | 0.000 |  | 0.000 |
| ROC: Harell’s C statistic | 0.779 (0.769, 0.789) |  | 0.718 (0.706, 0.729) |  |
| *Available post-delivery  ^Assume suspected macrosomia available pre-delivery (information available from actual birthweight); and that induction is usually decided pre partum  N/A: information not available to clinicians pre-delivery and so variable not included in model selection process  Blank: variable not selected in model selection process  For continuous variables ORs are mean and variance standardised (z-scores) | | | | |
